# Event and Comment

**Published:** 1918-01

**Authors:** 


### DENTAL REGISTER

Vol. LXXII JANUARY, 1918 No. 1

### EVENT AND COMMENT

**Ohio State Annual Meeting** By a unanimous verdict the meeting in Cleveland, December 4, 5, and 6, should be voted the best meeting ever held by this society, and this in spite of the troublous times, which would ordinarily interfere seriously with the success of a purely voluntary attendance at a dental convention. The attendance was larger than is usual and the published program was given entire with a single exception.

It is always difficult to carry out a program in a large city without annoying delays, but the officers insisted on proceeding by the clock, and considering that the city of Cleveland works on Eastern time, we had to get up early and have our meals promptly or miss much of the good material that was passing in a steady stream all the time. It had to be done this way or they never could have put through such a program in three days.

A new method of conducting the literary program was tried out this year. No discussions were allowed on any of the papers, and the essayists were each given one hour in which to present his subject. By the arrangement of the subjects on the program a variety of topics was presented at each session that relieved what might otherwise have been tiresome sessions. We are not sure that it is desirable to omit all free discussions, but in this case the different topics were so ably presented, mostly by specialists, that there did not seem to be much desire to take issue with the essayists.

Another innovation was the "Seminar" conducted by Dr. W. A. Price and eight specialists on the scientific problems involved in the treatment of infected pulpless teeth. Each leader gave a statement and demonstration of what could be done, for instance; in the way of definitely sterilizing infected dentin and cementum; or in sterilizing the apical region by ionic medication; or what clinical evidence can be had to clear the diagnosis for saving or extracting teeth, etc. All of the ten subjects presented were treated scientifically and so far as our present knowledge permits, with satisfactory conclusions. This seminar was a whole dental society program in itself, and was original so far as we know. It might be called a combination of the essay and clinic form of presentation, not so formal or technical, and because of its sequential order of presentation a very effective elucidation of what we ought to know about this most important subject so far as scientific research and clinical demonstrations have revealed accurate knowledge to us.

The clinical program was given in one session in the form of "Progressive Clinics." These clinics, eight in number, were mostly on the technical handling of pulpless canals from the various standpoints of accurate and safe fillings. The clinics were all that could have been asked from the standpoint of their adequate presentations except that the time allowed to each was so short that the clinicians could not do themselves justice because they were so hurried and the audience did not have the opportunity to assimilate the material and ideas presented. This method has much in it to commend, but it will never be successfully done until more time is given for the presentation of each subject, and the clinicians come to the clinics prepared to say and do what they undertake in the most precise manner. Too many unnecessary apologies and useless technical explanations, to say nothing of irrelevant remarks and interruptions by the audience make this form of clinic disappointing and valueless. Anyone undertaking a clinic of this kind should have well prepared models and charts showing exactly and only the things that can't easily be put into words, and then he should have several rehearsals of the clinic, before a good critic, before attempting it in public.

The exhibits by the dealers was unusually large, but because of the hotel accommodations, not so well displayed as at Dayton last year. There were a number of new appliances and materials and the exhibits were crowded with visitors at every opportunity.

A fraternal supper, not a fraternity, was held in one of the hotels one evening which was a real love feast and gave opportunity for much display of good-fellowship and the only chance for relaxation from the more serious affairs of the convention.

The last afternoon was exhibitors' session, and opportunity to visit the Research Institute and the new building for the Dental Department of Western Reserve University. The weather was not favorable to city sight seeing and many other interesting events had to be omitted. The hospitality of the city, the hotels, and the Cleveland dentists was all that the most exacting could ask. The program committee and the other officers can certainly feel well repaid for all the hard work they must have done to provide so excellent a meeting. It requires much courage and effort in these strenuous times to leave home even for so necessary an event as a meeting of this kind, but we are sure it paid, and all who attended will be and do better the coming year because they went to this meeting.

**The Western Dental Journal Ceases Publication**

The publishers of this well known and valued journal announce that with the December issue they will discontinue publication. No reason is given for this action, and we are sure the profession in the West will deeply regret this event. This journal has had an honorable career and served the profession in a most creditable manner for over thirty years. Like all such efforts on the part of self-sacrificing editors and publishers it probably has not received the appreciative support needed to hearten the publishers or to stimulate the editor to the highest type of efficiency. Like many other editors and publishers they have decided to spend their talents and funds where they will be more appreciated. There certainly must be a sphere of usefulness for a dental journal in that part of our country and we are surprised that with all the enterprising talent in that section that this journal should not be an actual necessity. The profession needs just such an organ and will not long do without one, and why would it not have been better to keep the one they have, and by a more united effort make it represent in a more complete way the vitality of professional activity in that section?

**National Dental Association Meeting for 1918**

The officers have already announced the dates for the next meeting of the National Society, which will be held in Chicago, August 5 to 9, 1918, in the Auditorium Hotel. Big plans are already under way for the meeting, among them will be the dedication of a memorial monument to Dr. G. V. Black. With Major Logan as president and Dr. J. P. Buckley as chairman of the publicity committee there certainly will be some meeting of dentists in Chicago next year, unless the ''Kaiser'' puts unexpected difficulties in the way. A society with 20,000 members and a record of over 5,000 in attendance at its last meeting in New York there will certainly be some stimulant for the Chicago men to make good, whatever may be the war situation. It will be some contrast we anticipate with the meeting of the old American Association which held a meeting in Chicago in 1865, the last year of our Civil War, when there were 124 dentists all told to respond to roll call. They were, however, the men who put dentistry on a substantial professional basis, and started it on a career that has culminated in an act of Congress which gives to dentistry the same legal status as that given to the medical profession, also in this year of a war to put all humanity on an equal basis.

**The Black Memorial** The committee authorized to collect money for a memorial monument for Dr. G. V. Black reported at the National Convention in New York that over $7,500 had been subscribed up to that date. It is not definitely known where the memorial will be located or what form it may take. A committee of three gentlemen of artistic reputation has been appointed to select the design most appropriate. It is hoped it may be completed in time to be dedicated next August at the time of the national meeting in Chicago. It would be most appropriate if it could be associated in some practical way with the work for which he gave his life and to which he was so sincerely devoted. If the dentists of Chicago and the Middle West could contribute a sufficiently large fund to build a suitable annex to the Chicago Library, or the Art Institute, or the New Field Museum, to house a Dental Museum and Library with facilities for scientific research, this would carry on perpetually the work which our profession needs more than all else, and this would surely gratify the desires of all those who knew and loved Dr. Block, and undoubtedly would meet his approval if he could know of it, better than anything else.

**Research Institute** The report of Dr. Price to the National Dental Association as to the work of the Research Institute reveals the fact that the property value of the buildings, ground and equipment is conservatively appraised at $60,000, on which $28,500

has been paid, leaving a mortgage balance of $32,500. Over $32,000 is subscribed to the Research Fund, and over $5,000 to the Building Fund. There is $1,200 in current expense funds. This is a most hopeful financial showing. During the next year there will be available for carrying on research work, for salaries, etc., over $15,000 derived from the extra dues from the state societies. $8,675.23 was paid out last year as salaries for research workers. During the coming year the work of research at the Institute will be confined largely to the pathologic problems that are at present occupying so much of the professions' attention. The directors are on the lookout for a suitable man to prosecute this research. It is the expectation of Dr. Price that he will very soon have a man at work on this problem who is capable of working out the fundamentals for this entire subject.

